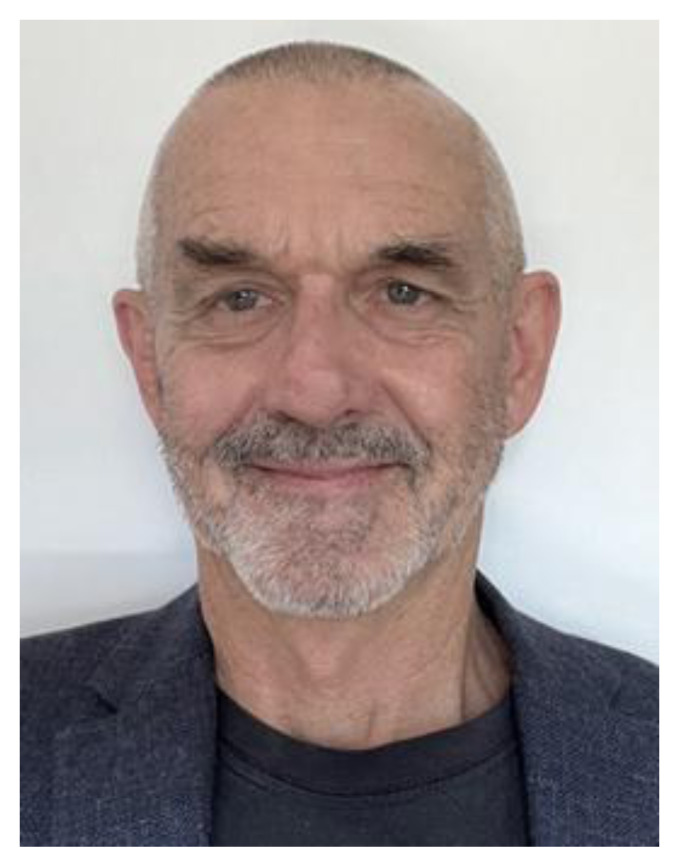# Reflecting on 2024 and Looking Ahead

**DOI:** 10.17159/2078-516X/2025/v37i1a20982

**Published:** 2025-02-15

**Authors:** Mike Lambert

**Affiliations:** Editor-in-chief

At the beginning of 2025, we at the South African Journal of Sports Medicine (SAJSM) would like to take this opportunity to reflect on a successful 2024. The journal flourishes due to the unwavering support of contributors, reviewers, readers, and the frameworks that support our operations. It has been a year of growth, challenges, and achievements reaffirming our commitment to advancing sports medicine research in South Africa and beyond. One of the most significant achievements of 2024 was the seamless integration of our published papers into PubMed. This marks an important step in ensuring that the high-quality research disseminated through SAJSM reaches a wider international audience, enhancing visibility and impact.

Our acceptance rate of 26% reflects the rigorous peer-review process that upholds the journal’s standards. This selectivity ensures that we publish research of the highest quality and relevance to sports medicine.

In terms of readership, SAJSM continues to attract a geographically diverse audience. While most visits to the journal website are from South Africa, we also see significant engagement from Singapore, the USA, and India. This highlights the growing international recognition of SAJSM as a valuable resource for sports medicine professionals.

April, 2024 emerged as the busiest month for paper views, with 5,510 views, contributing to an impressive total of 45,614 views for the year. Notably, the paper “*The influence of psychological readiness of athletes when returning to sport after injury*” ^[1]^ garnered the highest number of views (697 views), followed by “*Comparing cardiorespiratory fitness and physical activity levels between third- and fifth-year medical students in a South African university*”^[2]^ (616 views). These statistics highlight the diversity of topics covered by the journal and the relevance of the research to our readership.

Finding qualified and willing reviewers remains a persistent challenge for SAJSM and many other journals. In 2024, we took proactive steps to address this by hosting courses on peer review at the University of Cape Town, the University of the Western Cape, Rhodes University, and the University of Pretoria. These initiatives were fruitful, with several attendees providing exemplary reviewer reports. The peer review training supports the journal and contributes to building capacity in the academic community.

The introduction of print charges has not deterred submissions, which is promising. These fees play a crucial role in covering the journal’s operational costs, including making our publications accessible on PubMed. Significantly, we continue to receive support from the Academy of Science of South Africa (ASSAf). Their guidance ensures that SAJSM remains at the forefront of open-source publishing and meets the Department of Higher Education’s rigorous accreditation standards.

Integrating artificial intelligence (AI) tools has become increasingly significant in the rapidly evolving landscape of academic publishing. At SAJSM, we use AI to enhance the integrity of our publishing process. AI tools help us assess the degree of similarity in submitted papers, ensuring that only those with low similarity indices proceed to review. This has effectively identified instances of plagiarism and maintained the ethical standards expected of a reputable journal. However, we remain vigilant about the potential misuse of AI in academic publishing. While we embrace its benefits, we are committed to ensuring that AI is applied responsibly and does not compromise the authenticity or originality of research.

The field of sports medicine is dynamic, and we strive to ensure that SAJSM evolves to meet its changing needs. In 2025, we look forward to a special edition focusing on tennis-related topics. This will feature outputs from the Society of Tennis Medicine and Science’s conference in the Netherlands in April 2025, showcasing cutting-edge research in this niche area of sports medicine.

As we move into 2025, we remain committed to fostering a platform for high-quality research, addressing emerging trends, and supporting the local and global sports medicine community. Our achievements in 2024 would not have been possible without the collective efforts of our contributors, reviewers, and the ASSAf framework. We thank everyone who has supported SAJSM and look forward to another year of collaboration and innovation.

Here’s to a promising 2025, filled with new opportunities, impactful research, and continued growth for the South African Journal of Sports Medicine.[Fig f1-2078-516x-37-v37i1a20982]

**Figure f1-2078-516x-37-v37i1a20982:**